# Exploration of the methods of establishing the minimum clinical important difference based on anchors and their applications in the quality of life measurement scale QLICP-BR (V2.0) for breast cancer

**DOI:** 10.3389/fonc.2023.1123258

**Published:** 2023-03-27

**Authors:** Xuan Zhou, Yuxi Liu, Jianfeng Tan, Liren Hu, Huanwei Chen, Chonghua Wan

**Affiliations:** ^1^ Key Laboratory for Quality of Life and Psychological assessment and Intervention, School of Humanities and Management, Research Center for Quality of Life and Applied Psychology, Guangdong Medical University, Dongguan, China; ^2^ School of Public Health, Guangdong Medical University, Dongguan, China; ^3^ Oncology Department, Central Hospital of Guangdong Nongken, Zhanjiang, China

**Keywords:** breast cancer, minimum clinically important difference, quality of life, QLICP-BR(V2.0), anchor-based methods, ROC curve method, multiple linear regression model

## Abstract

**Objective:**

The measurement of the quality of life (QOL) in patients with breast cancer can evaluate the therapeutic effects of medical treatments and help to provide reference for clinical decisions. The minimum clinically important difference (MCID) can be better used in clinical interpretation than the traditional statistical significance. Based on the anchors, a variety of ways including traditional and updated anchor-based methods were used to explore most suitable MCID, so that to find better interpretation on scores of the scale QLICP-BR(V2.0) (Quality of Life Instruments for Cancer Patients-Breast cancer).

**Methods:**

According to the investigation data of breast cancer patients before and after treatment, the most relevant indicators in various domains of QLICP-BR (V2.0) was found as an anchor to statistically analyze the value of MCID, and three analysis methods of anchors were used: Traditional anchor-based method, ROC curve method, multiple linear regression model analysis. Anchors are divided into four standards according to the degree of change in the treatment effect: one grade difference (Standard A), at least one grade difference (Standard B), one grade better (Standard C), better (Standard D). The final MCID value is selected from different statistical methods and classification standards that are most suitable for clinicians to use.

**Results:**

Using Q29 of the EORTC QLQ-C30 as an anchor has the highest correlation with each domain of QLICP. The order of magnitude of MCID values among the four standard groups is: standard A< Standard C< Standard B< Standard D. The MCID value obtained by the ROC curve method is the most stable and is least affected by the sample size, and the MCID value obtained by the multiple linear regression model is the least. After comparisons and discussions, Standard C in the multiple linear regression model is used to determine the final MCID, which is the closest to other methods. After integer the MCID values of Physical domain (PHD), Psychological domain (PSD), Social domain (SOD), Common symptoms and side effect domain (SSD), Core/general module (CGD), Specific domain (SPD), Total score(TOT) can be taken as 15,10, 10, 11, 10, 9 and 9, respectively.

**Conclusion:**

In the evaluation of the QOL of breast cancer patients, although the results of MCID values produced by different methods are different, the results are relatively close. The anchor-based methods make the results of MCID more clinically interpretable by introducing clinical variables, and clinicians and researchers can choose the appropriate method according to the research purpose.

## 1 Introduction

Female breast cancer has surpassed lung cancer as the most commonly diagnosed cancer in 2020 in the world (1). In China, 0.42 million women were diagnosed with breast cancer in 2020, accounting for about 18% of global female breast cancer. Breast cancer diagnosis rate may increase (2) with some potential influencing factors such as declining fertility rate, delayed first birth time, shorter breastfeeding time. The disease burden of different cancers is different. According to the Surveillance, Epidemiology, and End Results (SEER) database 5-year-survival rate of breast cancer was 90.3% from 2011 to 2017. In China the rate of disability-adjusted life years(DALYs) caused by female breast cancer has not changed obviously over the past 20 years, but the burden of it will increase for population aging. In 2019, the DALYs for breast cancer in China was 2.88 million accounting for 14.2% of the global burden (3). More attention should be paid to improving the QOL of women who have just been diagnosed with breast cancer or have completed initial treatment for breast cancer.

The scales used to measure the QOL of breast cancer patients commonly include Functional Assessment of Cancer Therapy-Breast (FACT-B) in the United States (4–6), Quality of Life Questionnaire-C30 (QLQ-C30) and the breast cancer-specific module QLQ-BR23 developed by European Organization for Research and Treatment of Cancer Quality of Life Questionnaires (EORTC) (7–10). Although the Chinese versions of QLQ-C30 and QLQ-BR23 and also FACT-B can be used for Chinese patients, they are lacking Chinese cultural backgrounds to some extent. For example, the QOL scales developed abroad are constructed mainly under the Western cultural system, which are more concern about the two aspects of religious belief and sexual life. In contrast, Taoism and traditional medicine focus on good temper and high spirit, good appetite and sleep are highly regarded in daily life with food culture being very important in China. Therefore, the breast cancer-specific scale QLICP-BR (Quality of Life Instruments for Cancer Patients-Breast cancer) was developed by Wan’s team in China (11–13). The QLICP-BR is one of a series of quality of life measurement scale systems (QLICP) independently developed by referring to QLQ-BR, FACT-B and considering Chinese culture. The updated version of QLICP-BR(V2.0) have good reliability and validity with clear hierarchical structure: items→ facets→ domains→ overall (11). Like FACT and QLQ-30, QLICP-BR(V2.0) also has a general module and a specific module, but it has some items regarding to Chinese culture in the general module such as appetite, sleep, energy, family support because Chinese culture pays more attention to the family relationship, eating and food, good temper and high spirit. The QLICP-BR(V2.0) can be used to measure various types of breast cancer patients in different time periods such as onset, treatment or rehabilitation period with good psychometrics (12, 13).

In the past, it was necessary to use *P* value to evaluate the changes of QOL scores before and after treatment. *P*< 0.05 means that there was a statistically significant difference, but this is more likely to happen with the increase of sample size. Clinicians also realize that when there was statistically significant difference in the calculated score, it does not represent the actual application in clinical treatment where they need a more accurate value to transform the degree of change in. Biostatisticians and epidemiologists have long advocated the use of confidence intervals (CIs) to replace or supplement *P* values (14). Although there are some methods such as equivalence test, non-inferiority test can be used to confirm the effects of the treatments in clinical researches, up to now the preferred indicator is minimal clinically significant difference (MCID) for it provides more powerful evidence for the interpretation of clinical conclusions, and it reflects the clinical significance of the smallest change in scores (15–17).

There are two traditional methods for calculating MCID, including anchor-based methods and distribution-based methods. The advantage of anchor-based methods is that clinical indicators can be used as anchors for calculating MCID, but the disadvantage is that it is difficult to find a suitable anchor having higher correlation with scores. Distribution-based methods can be directly calculated by various formulas, but the results obtained are difficult to apply clinically, and its specific practical significance is not easy to explain. According to the suggestion of the US Food and Drug Administration, the distribution-based methods should be used as an assistant to anchor-based methods (18). Only when the anchor-based methods cannot be implemented, the distribution-based methods can be used alone. Our research team used these two methods to analyze the QOL of breast cancer patients, and adopted traditional methods such as a traditional anchor-based method and three distribution-based methods (11).

MCID given by the distribution-based methods is more like a range. What we want is to screen MCID that is closer to clinical application, so we need to further explore the anchor-based methods to obtain MCID. In recent years, some new anchor-based methods have been put forward by scholars, such as Receiver Operating Characteristic (ROC) curve method and Multiple Linear Regression Model method (19–22). However, it is seldom used to evaluate the QOL of patients, especially cancer patients. The reliability of these methods for making MCID needs to be evaluated, and whether they can be reasonably explained in clinical application needs further discussion. Therefore, in this paper, we try to use advanced anchor-based methods to calculate MCID in QLICP-BR (V2.0), compared with traditional methods.

## 2 Materials and methods

### 2.1 Instruments

Patients enrolled in the study were asked to fill out the QLICP-BR (V2.0) and QLQ-C30 scales. The QLICP-BR (V2.0) was a scale of the QLICP system, and comprised 42 items with 32 items came from the general module QLICP-GM (V2.0) and 10 items from the module specific to breast cancer (SPD). The domains in QLICP-BR (V2.0) are: Physical domain (PHD), Psychological domain (PSD), Social domain (SOD), Common symptoms and side effect domain (SSD), Specific domain (SPD). The first four domains constitute the Core/general module (CGD), and the last domain SPD is for breast cancer disease. The scoring of QLICP-BR (V2.0) scale were based on the sum of the raw scores (RS) of the items to obtain each domain and the total score of the scale. All items form a total score (TOT). Each item of the QLICP-BR (V2.0) was rated on the 5-point Likert scale ranging from “not at all” to “extremely”. After reverse scoring of negatively worded items, each domain score was obtained as the total of the corresponding item responses. In order to compare with each other, the RS were converted into standard score (SS) (0–100) by range method, a linear transformation (11, 23). The conversion formula is as follows:


SS=(RS−Min)×100/R·R=Max−Min

The QLQ-C30 scale is a 30-item cancer-specific scale, which comprises of fifteen domains, including five functional subscales (physical, role, emotional, cognitive, and social), three multi-item symptom subscales (fatigue, pain and nausea/vomiting), a global health/quality of life subscales, and six single items addressing various symptoms and perceived financial impact. All items use a 4-point scale, namely, not at all, a little, quite a bit, and very much, except the global health status/QOL (Q29 and Q30) of which a 7-point scale is used.

### 2.2 Survey methods

All cases of breast cancer came from three hospitals: Affiliated Hospital of Guangdong Medical University, Center Hospital of Guangdong Nongken affiliated to Guangdong Medical University, Yunnan Cancer Hospital. Trained researchers screened hospitalized female breast cancer patients according to inclusion and exclusion criteria, and they were examined by breast doctors. Inclusion criteria: (1) Patients diagnosed with breast cancer after pathological examination; (2) Being able to independently read the scale and complete the answer; (3) Volunteering and agree to participate in this study. Exclusion criteria: (1) Patients with mental illness or other cognitive impairment. (2) With other cancers or metastases. (3) With critical illness.

According to the empirical method, the sample size required for investigation research is usually 5-10 times that of the items in the survey scale. Because there are 42 items in the scale, the sample size should be between 210 to 420.

The study protocol and the informed consent form were approved by the IRB (institutional review board) of the investigators’ institutions and the hospitals. After explaining the research purpose and obtaining their consent, the selected patients were investigated. The patients were asked to fill in the instruments (the QLICP-BR (V2.0) and QLQ-C30) at the time of admission to the hospitals by themselves independently. All patients available at the scheduled assessment time-point completed the measures at discharge to evaluate responsiveness, after approximately 4 weeks of treatment. Answers were checked immediately each time by the investigators in order to ensure its integrality.

### 2.3 Traditional anchor−based methods

Traditional anchor-based methods can be divided into two types: the anchor of the cross-section and the anchor of the longitudinal study (24). The anchor of the cross-section is independent of time and is usually used to detect multiple groups of experimental subjects at the same time. The longitudinal anchor is an index before and after the test to observe the difference, and an objective index such as a clinical examination index or a subjective evaluation index can be used. In this paper, we compared three anchors of Q29 item (how do you evaluate your overall health in the past week)? and Q30 item (how do you evaluate your overall quality of life in the past week)? of the QLQ-C30 and treatment effects to find which was more suitable for calculating MCID of the QLICP-BR(V2.0) domains. Treatment effect was a five-grade scoring index, which was comprehensively evaluated by doctors. The correlation coefficient of the test was considered to be no less than 0.3-0.35 (25). Changes in anchors before and after treatment were used to calculate MCID and were divided into four different standards: one grade difference (Standard A), at least one grade difference (Standard B), one grade better (Standard C), better (Standard D). We use *D_change_* to express the change of anchor value in each standards, and the average of the *D_change_* is the MCID, *D_change_* = *X*_1_-*X*_0_, *X*_0_ represents the patient’s baseline score (the day of admission) and *X*_1_ represents the patient’s score after treatment (the day before discharge).

### 2.4 ROC curve method

The principle of the ROC curve method is the same as the design of the diagnostic test, which is a new method for calculating MCID, and it also needs an index as an anchor. As in disease diagnosis, measured metrics are used to differentiate judgment anchor, such as improvement or no change, we still use the previous four standard anchors for ROC analysis. The calculated results include sensitivity and specificity, and then the ROC curve is drawn with sensitivity as the ordinate and 1-specificity as the abscissa. The value of MCID is to find the largest point corresponding to the Youden index (Sensitivity + specificity -1) on the ROC curve (26). The area under the curve(AUC) is to judge the accuracy of the cut-off value. As a general rule, AUC>0.7 means higher accuracy (27).

### 2.5 Multiple linear regression model

The method of linear regression is to estimate the MCID by establishing a regression equation model between the anchor and the target measurement index. The principle of the model is to take the changed scale score (*D_change_*) before and after treatment as the dependent variable, and take anchor grouping, baseline score and demographic variables as the independent variables to fit the regression equation (28). Demographic variables included age and education as confounding factors to adjust the model, and other variables were not included in the model due to the small number of samples in the classification. The regression model was as follows:


$$D_{change}=a_{0}+a_{1}x_{1}+a_{2}x_{2}+\cdot \cdot \cdot +a_{k}x_{k}$$

*a*_0_ is the constant term of the equation, *a_k_* is the coefficient of *x_k_*, *x*_1_: age, *x*_2_: education, *x*_3_: anchor grouping, *x*_4_: baseline score. After the linear regression equation is calculated, the predicted value can be output, and the mean of the predicted value is the MCID (26). The method of finding MCID by linear regression model can calculate a 95% confidence interval, and also can better adjust for potential confounding factors.

### 2.6 Data and statistical analysis

All data were entered into the database by Epidata 3.0, and statistical analysis was made by SPSS 26.0 software. Cronbach coefficient was used to evaluate the reliability of the scale, and descriptive statistical analysis was made on the basic demographic and clinical disease characteristics of patients, and the estimation of MCID was calculated by above corresponding methods.

## 3 Results

A total of 232 female breast cancer patients were included in this study. The average age of these patients was 50.02 ± 10.43 years, and 225 (96.98%) were married. Clinicians divided 206 cancer patients into four stages according to TNM, including 52 (25.24%) patients in stage I, 84(40.78%) patients in stage II, 44(23.36%) patients in stage III and 26(12.62%) patients in stage IV, and other 26 patients whose TNM stages were not reported. After treatments, 16(6.90%) patients were cured, 50(21.55%) patients got best, 152(65.52%) patients got better, 6(2.59%) patients had no change, 2(0.86%) patients got worse, and 6(2.59%) patients got worst.

### 3.1 Reliability and correlation analysis

Cronbach’s alpha of the QLQ-C30 is 0.812, and that of the QLICP-BR (V2.0) is 0.890. Therefore, the reliability of these two scales is greater than 0.7 proving that both scales are credible. A Pearson correlation analysis was carried out between each domain of the QLICP-BR(V2.0) and Q29, Q30, treatment effects, respectively (**Table 1**). It can be found that the correlation coefficient between each domain of the QLICP-BR (V2.0) and Q29 is greater than 0.30 except of SSD domain (*r*=0.25). It also found that the correlation coefficient between each domain of the QLICP-BR (V2.0) and Q30 as well as treatment effects are lower than that with Q29, so it was more reasonable to use Q29 as the anchor for MCID by traditional anchor−based method.

**Table 1:** Correlation coefficients between Q29,Q30, treatment effects and domains of the QLICP-BR (V2.0).

Item	PHD	PSD	SOD	SSD	CGD	SPD	TOT
Q29	0.34^**^	0.69^**^	0.46^**^	0.25^**^	0.58^**^	0.39^**^	0.66^**^
Q30	0.27^**^	0.67^**^	0.40^**^	0.18^**^	0.51^**^	0.37^**^	0.59^**^
treatment effects	0.10	0.01	-0.04	0.10	0.10	0.11	0.08

PHD, physical domain; PSD, psychological domain; SOD, social domain; SSD, common symptoms and side effect domain; CGD, core/general module; SPD, specific domain; TOT, the total. ^**^P< 0.01.

### 3.2 MCID by traditional anchor−based method

Among the respondents who answered Q29 questions twice before and after treatments, 66(28.45%) people felt that their overall health status had not changed in the past week, while the sample sizes of the other four criteria were Standard A 116(50.00%) people, Standard B 166(71.55%) people, Standard C 90(38.79%) people and standard D136(58.62%) people. The *D_change_* were calculated for all four standard groups, and the results of the mean and standard deviation are shown in **Table 2**. The mean in traditional anchor-based methods represents the MCID value of the groups. It can be observed from the results that all MCID values in the four standards are in the order of size Standard A< Standard C< Standard B< Standard D, and the value of MCID ranges from 10.69-19.97. No matter which standard is used for grouping, the MCID of the PHD is always the largest, while the change of breast cancer SPD is always the smallest.

**Table 2:** The MCID of QLICP-BR (V2.0) determined by traditional anchor-based methods under different standards (*n*_A_=116, *n*_B_=166, *n*_C_=90, *n*_D_=136).

Domain	Items	$\bar{x}+s$				MCID			
		Standard A	Standard B	Standard C	Standard D	Standard A	Standard B	Standard C	Standard D
PHD	8	16.24 ± 10.65	18.88 ± 12.31	17.15 ± 11.28	19.97 ± 12.68	16.24	18.88	17.15	19.97
PSD	9	11.37 ± 9.24	15.14 ± 12.51	12.07 ± 9.06	16.09 ± 12.52	11.37	15.14	12.07	16.09
SOD	8	11.31 ± 8.08	14.10 ± 13.80	12.60 ± 8.15	15.58 ± 14.54	11.31	14.10	12.60	15.58
SSD	7	12.07 ± 8.69	14.59 ± 12.08	12.62 ± 8.60	15.15 ± 11.59	12.07	14.59	12.62	15.15
CGD	32	11.49 ± 7.41	13.93 ± 9.26	12.40 ± 7.44	14.90 ± 8.92	11.49	13.93	12.40	14.90
SPD	10	10.69 ± 8.34	12.17 ± 9.83	11.78 ± 8.55	13.16 ± 9.94	10.69	12.17	11.78	13.16
TOT	42	11.23 ± 7.47	14.24 ± 9.63	12.70 ± 7.39	15.69 ± 9.30	11.23	14.24	12.70	15.69

PHD, physical domain; PSD, psychological domain; SOD, social domain; SSD, common symptoms and side effect domain; CGD, core/general module; SPD, specific domain; TOT, the total.

### 3.3 MCID by ROC curve method

**Figure 1:**
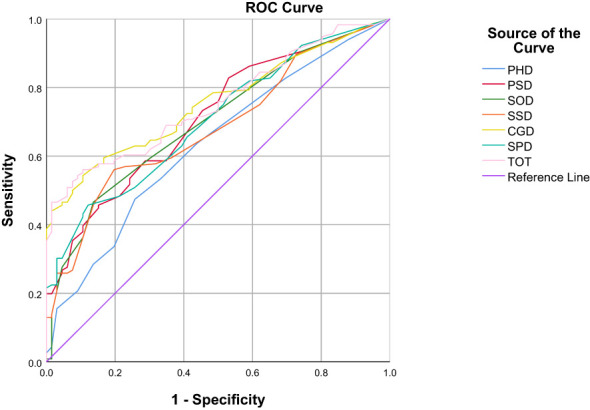
ROC curves of various domains under the Standard A.

**Figure 2:**
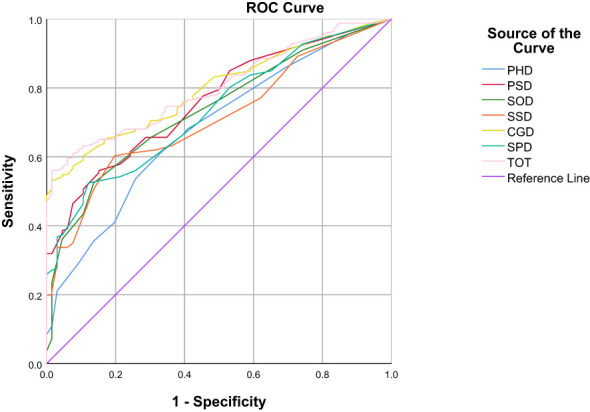
ROC curves of various domains under the Standard B.

**Figure 3:**
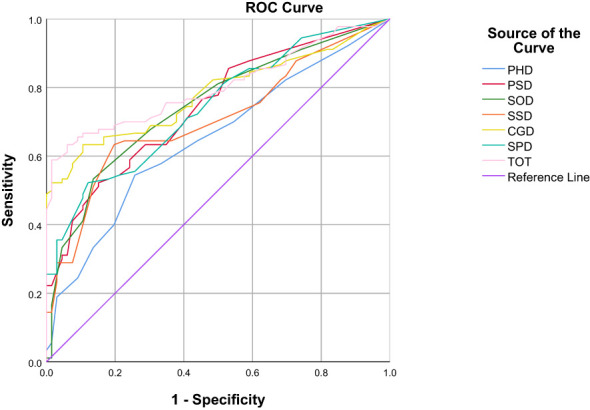
ROC curves of various domains under the Standard C.

**Figure 4:**
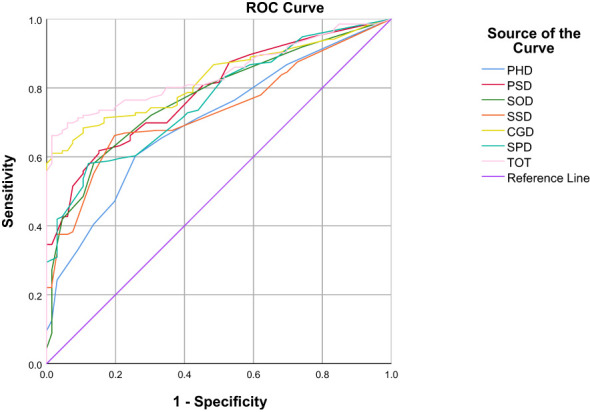
ROC curves of various domains under the Standard D.

In the ROC curve analysis, the four groups of data changed in the anchor-based methods are used as state variable 1, and the unchanged group is used as state variable 0, and various domains are included as test variables into the ROC curve for analysis (**Table 3**; **Figures 1**–**4**). The AUC values in all domains are greater than 0.7 except for all PHD (0.63-0.68) and SSD (0.68) under standard A, which indicates that the ROC curve method to calculate the value of MCID is credible. Although the grouping criteria are different, many MCID values calculated according to the ROC curve method are the same among different groups. The MCID value of all PSD domains are 11.11, all SOD domains are 10.94, all SSD domains are 10.71, and all SPD domains are 10.0. In all domains, the MCID value of PHD was 3-6 points higher than that of other domains.

**Table 3:** The MCID of QLICP-BR (V2.0) determined by ROC curve method under different standards (*n*_A_=182, *n*_B_=232, *n*_C_=156, *n*_D_=202).

Domain	AUC				MCID			
	Standard A	Standard B	Standard C	Standard D	Standard A	Standard B	Standard C	Standard D
PHD	0.63	0.68	0.65	0.65	17.19	14.06	17.19	17.19
PSD	0.71	0.76	0.74	0.74	11.11	11.11	11.11	11.11
SOD	0.70	0.74	0.75	0.75	10.94	10.94	10.94	10.94
SSD	0.68	0.71	0.71	0.71	10.71	10.71	10.71	10.71
CGD	0.75	0.80	0.78	0.78	10.00	13.00	10.00	10.00
SPD	0.70	0.73	0.74	0.74	10.00	10.00	10.00	10.00
TOT	0.75	0.80	0.80	0.80s	8.59	12.50	12.50	12.50

PHD, physical domain; PSD, psychological domain; SOD, social domain; SSD, common symptoms and side effect domain; CGD, core/general module; SPD, specific domain; TOT, the total.

### 3.4 MCID by multiple linear regression model

In order to facilitate the comparison between each other, MCID values are calculated in groups of four standards (**Tables 4**–**7**). In the multiple linear regression model with each *D_change_* as the dependent variable and four independent variables, after statistical analysis, all models have statistical significance. The size of MCID is the same as that calculated by anchor-based methods, and the order is standard A< Standard C< Standard B< Standard D. The MCID value of the PHD domain ranges from 14 to 17 points, which is still the one that needs the most change among all domains. The maximum value of R^2^ is 0.53, although most of them are not too high, which means that the requirements for independent variables should be more cautious when selecting the MCID calculated by multiple linear regression model.

**Table 4:** The MCID of QLICP-BR (V2.0) determined by multiple linear regression models under Standard A (*n*_A_=182).

Domain	MCID	R^2^	Multiple linear regression model
Physical domain	14.46	0.12	$D_{change}=26.41-0.12X_{1}+1.47X_{2}+3.53X_{3}-0.16X_{4}$
Psychological domain	9.28	0.14	$D_{change}=7.75-0.01X_{1}+1.28X_{2}+5.52X_{3}-0.06X_{4}$
Social domain	9.36	0.13	$D_{change}=-0.88+0.04X_{1}+0.96X_{2}+5.32X_{3}-0.04X_{4}$
Common symptoms and side effect domain	10.16	0.24	$D_{change}=23.44+0.08X_{1}+1.08X_{2}+3.98X_{3}-0.26X_{4}$
Core/general module	9.24	0.24	$D_{change}=13.20-0.03X_{1}+1.24X_{2}+5.51X_{3}-0.12X_{4}$
Specific domain	8.65	0.43	$D_{change}=33.60+0.09X_{1}+1.41X_{2}+4.36X_{3}-0.42X_{4}$
Total	8.87	0.23	$D_{change}=2.40+0.02X_{1}+1.35X_{2}+6.19X_{3}-0.02X_{4}$

ALL Domain p<0.01.

**Table 5:** The MCID of QLICP-BR (V2.0) determined by multiple linear regression models under Standard B (*n*_B_=232).

Domain	MCID	R^2^	Multiple linear regression model
Physical domain	16.73	0.20	$D_{change}=35.35-0.04X_{1}+1.60X_{2}+5.04X_{3}-0.34X_{4}$
Psychological domain	12.43	0.22	$D_{change}=21.02-0.03X_{1}+1.02X_{2}+8.98X_{3}-0.27X_{4}$
Social domain	11.77	0.09	$D_{change}=3.31+0.08X_{1}+0.23X_{2}+8.18X_{3}-0.03X_{4}$
Common symptoms and side effect domain	12.38	0.36	$D_{change}=39.48+0.13X_{1}+0.73X_{2}+4.68X_{3}-0.46X_{4}$
Core/general module	11.47	0.28	$D_{change}=25.24-0.03X_{1}+1.04X_{2}+7.06X_{3}-0.31X_{4}$
Specific domain	10.15	0.47	$D_{change}=38.37+0.14X_{1}+1.09X_{2}+5.19X_{3}-0.49X_{4}$
Total	11.53	0.25	$D_{change}=16.11+0.06X_{1}+0.99X_{2}+8.50X_{3}-0.22X_{4}$

ALL Domain p<0.01.

**Table 6:** The MCID of QLICP-BR (V2.0) determined by multiple linear regression models under Standard C (*n*_C_=156).

Domain	MCID	R^2^	Multiple linear regression model
Physical domain	14.68	0.15	$D_{change}=26.18-0.12X_{1}+1.73X_{2}+4.09X_{3}-0.17X_{4}$
Psychological domain	9.33	0.21	$D_{change}=11.24-0.01X_{1}+1.55X_{2}+6.27X_{3}-0.12X_{4}$
Social domain	9.78	0.20	$D_{change}=3.82+0.03X_{1}+1.36X_{2}+6.33X_{3}-0.03X_{4}$
Common symptoms and side effect domain	10.16	0.30	$D_{change}=25.99+0.05X_{1}+1.38X_{2}+3.97X_{3}-0.28X_{4}$
Core/general module	9.39	0.33	$D_{change}=13.86-0.06X_{1}+1.41X_{2}+6.26X_{3}-0.11X_{4}$
Specific domain	8.94	0.51	$D_{change}=35.52+0.05X_{1}+1.32X_{2}+5.28X_{3}-0.41X_{4}$
Total	9.32	0.35	$D_{change}=4.05-0.02X_{1}+1.52X_{2}+7.46X_{3}-0.02X_{4}$

ALL Domain p<0.01.

**Table 7:** The MCID of QLICP-BR (V2.0) determined by multiple linear regression models under Standard D (*n*_D_=202).

Domain	MCID	R^2^	Multiple linear regression model
Physical domain	17.14	0.25	$D_{change}=39.07-0.06X_{1}+1.70X_{2}+5.22X_{3}-0.38X_{4}$
Psychological domain	12.67	0.31	$D_{change}=26.18-0.004X_{1}+1.05X_{2}+9.74X_{3}-0.327X_{4}$
Social domain	12.42	0.13	$D_{change}=8.28+0.06X_{1}+0.23X_{2}+9.49X_{3}-0.08X_{4}$
Common symptoms and side effect domain	12.43	0.48	$D_{change}=46.94+0.06X_{1}+0.82X_{2}+4.10X_{3}-0.50X_{4}$
Core/general module	11.76	0.36	$D_{change}=28.08-0.02X_{1}+1.03X_{2}+7.70X_{3}-0.32X_{4}$
Specific domain	10.52	0.53	$D_{change}=41.00+0.08X_{1}+0.90X_{2}+5.84X_{3}-0.49X_{4}$
Total	12.11	0.34	$D_{change}=18.77+0.01X_{1}+0.87X_{2}+9.78X_{3}-0.22X_{4}$

ALL Domain p<0.01.

## 4 Discussion

Although the names and abbreviations of the minimum differences are not completely consistent, such as Minimal clinical important difference (MCID), Minimal clinical important change (MCIC), Minimal important difference (MID), Minimal clinically meaningful changes (MCMC), Clinical significance changes (CSC), the application of MCID has been very extensive, especially in clinical field. In a large number of cancer treatment studies, the formulation of MCID has been applied to almost all cancers such as lung cancer, gastric cancer, breast cancer, prostate cancer, head and neck cancer, and esophageal cancer. It can be seen that MCID has certain clinical application value.

There are many analysis methods for MCID, and the commonly used analysis methods include anchor-based methods and distribution-based methods. The anchor-based methods analyzes by measuring the change of an objective or subjective index before and after treatment as an anchor, while the distribution-based methods does not require an anchor, and calculates through a statistical formula to obtain the MCID. The distribution-based methods can obtain the MCID value based on the baseline data, which is easier to calculate than the anchor-based methods. However, this method lacks the significance of combining with clinical, and the QOL of patients measured at different times and different states will be different, so the MCID value of the distribution-based methods may have a large change. Therefore, our study explored the QOL of breast cancer based on the traditional and updated anchor-based methods to formulate MCID. The anchors required in the anchor-based methods can be objective indicators such as treatment effect, clinical examination results (29), or subjective indicators such as the fatigue or pain in patients (30, 31).

There can be a single anchor or multiple anchors, but no matter which one is used in clinical applications, the following two requirements must be met: First, it must be clinically interpretable; otherwise doctors do not know how the MCID value is used to evaluate the treatment effect. Second, there must be a significant correlation between the target and the anchor. The stronger the correlation, the higher the credibility of this anchor-based methods explanation. A single anchor needs a higher degree of correlation to prove more reliable than multiple anchors (32). There are many studies using the QLQ-C30 questionnaire to calculate MCID, so we used Q29 and Q30 as anchors when analyzing the MCID of QLICP-BR (V2.0), and also compared the correlation of treatment effects. The results show that Q29 is suitable as an anchor, but unfortunately, the correlation of treatment effect is weak. In future studies, we will continue to seek other suitable objective indicators as anchors to analyze the MCID of breast cancer patients’ quality of life.

Data from anchor-based methods are easier to analyze, especially with larger sample sizes. We divided anchors into four standard groups based on clinically possible conditions and used them to explore suitable MCID. The results for Standard A and Standard B are reported in one of our other articles for the purpose of comparing the difference between the anchor-based methods and the distribution-based methods (11). The MCID value based on Standard A is the smallest, and the patient feels that there is a change, whether the change is better or worse. Because different standards or methods give different MCIDs, so if clinicians need the smallest MCID, then it can be analyzed and given according to Standard A. The MCID value of Standard B is about 1 higher than that of Standard A, so if clinicians want to achieve the minimum clinical treatment effect, they can use the method of Standard B to obtain the MCID. The MCID obtained by Standard D is the largest among all groups, which means that if all patients are to achieve clinical improvement, this standard can be sufficient to evaluate the actual clinical value of treatment. It can be seen that when the anchor-based method is used, the difference of MCID values of the four groups is greater than that when the other two methods are used.

ROC curve and multiple linear regression model are advanced methods derived from anchor-based methods. They are similar in that the sample sizes under their respective standards A-D are the same, but the difference is that multiple linear regression method combines the baseline data of patients, so it can better explain the clinical significance and reflect the actual clinical changes. The results of ROC curve method show that the MCID value is relatively stable, especially the results of Standard C and Standard D are exactly the same, which means that the method of ROC curve can be widely used and is less affected by changes in sample size. Turner (33) demonstrated by example that using the data of the entire cohort and the ROC curve to formulate MCID can effectively improve the accuracy of MCID. AUC should be greater than 0.7, which indicates that the diagnostic efficiency is good. The AUC score in PhD field is close to 0.7, and the MCID is greater than that in other fields. Compared with the results in anchor-based methods, the value is also very close. The method of multiple linear regression analysis of MCID has the advantage that it can be adjusted by potential variables. Clinicians first propose variables that can have a large impact on post-intervention outcomes, and then incorporate these variables into the model along with grouping variables and baseline data. The MCID of Standard A obtained by multiple linear regression model analysis is the smallest MCID value that can be obtained in this study. Considering the stratification factors and clinical application value, after comparing the anchor-based method, ROC curve method and multiple linear regression method, we decided to adopt the Standard C result in multiple linear regression model as the final value of MCID. For the convenience of use, the value of MCID can be an integer in clinical application so the MCID values of PHD, PSD, SOD, SSD, CGD, SPD, TOT can be taken as 15,10, 10, 11, 10, 9 and 9, respectively.

The MCID values obtained by the anchor-based methods were smaller than those obtained by the distribution-based methods, probably because the introduction of an anchor related to clinical treatment concretized the clinical significance of the results. Chan (34) used QLQ-C30 and Fatigue Symptom Inventory-Short Form (MFSI-SF) to measure the QOL of patients with breast cancer, and calculated the MCID, with the MCID based on anchor being 8.69, and that of distribution method being 5.39-10.79. Cheung (35) used QLQ-C30 and Assessment of Cancer Therapy Cognitive Function (FACT-Cog) to measure the QOL of patients with breast cancer, the MCID based on anchor was 9.6, and that of distribution method was 6.9-10.6. Eton (36) used FACT-B to test the quality of life of patients with breast cancer, preliminarily estimated the MCID by anchor method, and further narrowed the scope of MCID by effect sizes in distribution method. Although these scales are different, their standard scores are between 0-100 and are of comparability. According to studies on measuring the quality of life of breast cancer patients with different tools, it is necessary to calculate MCID based on anchor method first before distribution method.

In addition to the three statistical analysis methods we used above, some scholars also proposed to draw the cumulative density function plots based on the anchor-based methods to analyze MCID. However, this method currently does not have clear guidelines to make the selected points sufficiently convincing (16). The literature on MCID was reviewed in the early days of the study, but no information was found on the reported sample size of the study. Therefore, we used the common sample size (usually greater than 100) required by the survey scale when selecting the sample. The ROC curve method also analyzed that the MCID is less affected by the change of the sample size, so many people think that the *P* value is affected by the sample size, and the MCID can be used more to represent the results of the study. While an underestimated MCID may produce an overly optimistic estimate of the outcome of treatment, an overestimated MCID can judge an effective treatment regimen as ineffective (33, 37, 38). Therefore, we finally selected the MCID calculated by Standard C in the multiple linear regression analysis as the final result of the QOL evaluation of breast cancer patients by comparing three statistical analysis methods and different standard groups.

The disadvantage of this paper is that the final MCID value may be affected by the change of data structure of population samples, which is also a common problem of all anchor-based methods at present. In addition, the baseline data used for evaluation, such as demographic characteristics and disease diagnosis indicators, may be lacking. According to the above problems, some scholars have proposed that future research should focus more on understanding the changes of results caused by different statistical methods, and developed a reliability evaluation tool of minimal important differences for patient reported outcomes on anchor-based methods (39). Therefore, we may collect a large number of clinical verification results in the next research, use this tool to evaluate the reliability of MID, and provide a better and more reliable result to clinicians.

## Data availability statement

The raw data supporting the conclusions of this article will be made available by the authors, without undue reservation.

## Ethics statement

The studies involving human participants were reviewed and approved by the IRB (Institutional Review Board) of the affiliated hospital of Guangdong Medical University (PJ2012052, YJYS2019010). The patients/participants provided their written informed consent to participate in this study. Written informed consent was obtained from the individual(s) for the publication of any potentially identifiable images or data included in this article.

## Author contributions

CW conceived and designed the study. XZ, YL, JT, LH, and HC performed the data collection. XZ, YL, and XZ and YL analyzed data and drafted the manual. CW revised the manuscript deeply. All authors contributed to the article and approved the submitted version.
